# The Effect of pH on the Electrical Capacitance of Phosphatidylcholine–Phosphatidylserine System in Bilayer Lipid Membrane

**DOI:** 10.1007/s00232-014-9644-1

**Published:** 2014-02-28

**Authors:** Monika Naumowicz, Zbigniew Artur Figaszewski

**Affiliations:** 1 Institute of Chemistry, University of Bialystok, Al. J. Pilsudskiego 11/4, 15-443 Bialystok, Poland; 2 Laboratory of Electrochemical Power Sources Faculty of Chemistry, University of Warsaw, Pasteur St. 1, 02-093 Warsaw, Poland

**Keywords:** Electrochemical impedance spectroscopy pH, Acid–base equilibria, Bilayer membrane, Phosphatidylcholine, Phosphatidylserine

## Abstract

This paper reports measurements on the pH dependence of the electrical capacitance of lipid membranes formed by 1:1 phosphatidylcholine-phosphatidylserine mixtures. A theoretical model was developed to describe this dependence, in which the contributions of functional groups (as the active centers of adsorption of the hydrogen and hydroxide ions) to the overall membrane capacitance were assumed to be additive. The proposed model was verified experimentally using electrochemical impedance spectroscopy. The theoretical predictions agreed with the experimental results over the measured pH range. A minimum corresponding to the isoelectric point appeared in both the theoretical equation and the experimental data.

## Introduction

As their basic structural unit, biological membranes contain sheet-like assemblies of thousands of amphiphilic lipid molecules held together by hydrophobic interactions between their acyl chains. These lipid bilayers form the boundaries between the intracellular cytoplasm and the extracellular environment, as well as between the interior of many of the cell’s organelles and the cytoplasm (Peetla et al. 2009). Phosphatidylcholine (PC) is one of the major phospholipids in eukaryotic biological membranes constituting 40–60 % of the total phospholipid content (Kent 2005). Although zwitterionic and uncharged lipids are the predominant lipid components of eukaryotic cell membranes, anionic phospholipids are also essential structural and functional components of such membranes (Buckland and Wilton 2000; Lentz 1999). Phosphatidylserine (PS) is the major anionic phospholipid in eukaryotic membranes. One of its primary functions is to impart a negative charge to the inner surface of the membrane lipid bilayer. This negative surface charge is required for the binding and activation of various peripheral membrane proteins, including phospholipases (Buckland and Wilton 2000), myristoylated proteins (McLaughlin and Aderem 1995), and components of the blood coagulation process (Lentz 1999).

Researchers have studied the effects of the membrane surface charge on the conformation, orientation, and dynamics of the lipid polar headgroup at the PS bilayers surface (Browning 1981; Roux and Neuman 1986; Roux et al. 1989). Such studies suggest a relatively rigid structure for the PS headgroup compared to both PC and phosphatidylethanolamine (PE) (Browning 1981). The orientation and dynamics of the PS headgroups appear to be largely determined by the sign and magnitude of the surface charge of the bilayer, which, in turn, can be affected by the binding of soluble cationic peptides to the membrane surface and by the incorporation of a transmembrane peptide with cationic termini. These results can be rationalized by assuming that the orientation of the dipole of the polar headgroup is dependent on the magnitude and location of charges near the bilayer surface (Scherer and Seelig 1989). Using Fourier transform infrared (FTIR) spectroscopy, Lewis and McElhaney (2000) revealed that PS experiences increased hydrogen bonding compared to PC. Cevc et al. (1981) and MacDonald et al. (1976) studied the complex effects of pH, electrostatics, ion binding, and headgroup hydration for disaturated PS.

The artificial lipid membrane system has been employed extensively as an experimental model of biomembranes, including lipid vesicles, Langmuir–Blodgett monolayers, cast lipid films, bilayer lipid membranes, supported bilayer lipid membranes, etc. (Tien and Ottova 2001). Among these mimetic biomembrane model systems, bilayer lipid membranes play an important role in biological activity. The formation of lipid bilayers between two aqueous solutions allows electrodes to be placed in both aqueous solutions while in contact with the membrane, thereby permitting the use of electrochemical techniques to study the lipid bilayer properties. Electrochemical impedance spectroscopy (EIS) is a powerful method that has been used to study diverse electrochemical systems, including fuel cells (Mamlouk and Scott 2011), corrosion (Li et al. 2010), batteries (Andre et al. 2011), double – layer studies (Pospišil et al. 2010a), electron transfer reactions (Pospišil et al. 2010b), solid state electrochemistry (Grubač and Metikoš-Huković 2004), electrocrystallization of metals (Pospišil et al. 2010a), hydrogen adsorption presses on Pd electrodes (Duncan and Lasia 2007), and electrode porosity (Jurczakowski et al. 2004). EIS has been also applied in bioelectrochemistry applications, especially in studies conducted on biological and artificial lipid membranes (Karolins et al. 1998; Naumowicz and Figaszewski 2005, 2009; Ye et al. 2003).

The pH affects many membrane-mediated biological processes, such as cholesterol domain formation, membrane fusion, drug-liposomes interactions, and lipid membrane phase transitions (Zhou and Raphael 2007). Normally, extracellular fluids have a pH of ~7.4, and cells regulate their internal pH at ~7.0. However, in some situations, biological membranes are exposed to environments with other pH values. The mechanisms that cells utilize to maintain their membrane integrity are not well understood. Thus, characterizing and understanding the interactions between protons, hydroxide ions (OH^−^), and lipid membranes are important biophysical problems.

A previous study described the adsorption of hydrogen ions (H^+^) and OH^−^ at the surface of the PC layer (Naumowicz et al. 2013). The electrical capacitance of the PC bilayer was minimal around pH 4 and increased as the pH of the solution increased or decreased. Depending on the pH of the solution, the PC molecule existed in one of four forms: free or with adsorbed H^+^ and/or OH^−^. The assumed model, which was based on the additivity of the electrical capacitance values of the PC forms, agreed well with the experimental values. In the present work, PS was chosen for further study. PS has –COO^(−)^ and –N^(+)^H_3_ groups situated in close proximity to each another, with the –PO^(−)^ group spaced apart from them. The aim of this work was to determine the dependence of the electrical capacitance of the PC–PS membrane in the pH range of 2.52–7.0, which includes the isoelectric point of the analyzed membrane (pH ~4.2).

## Theory

The lipid bilayer formed by a PC–PS system contains positively and negatively charged groups; therefore, it can participate in equilibrium reactions with both H^+^ and OH^−^. PC brings –PO^(−)^ and –N^(+)^(CH_3_)_3_ groups to the overall area of the membrane, whereas PS provides –PO^(−)^, –COO^(−)^, and –N^(+)^H_3_ groups.

The pH dependence of the electrical capacitance of the PC–PS bilayer can be described in terms of acid–base equilibria. Uniformly distributed active centers, where the H^+^ and OH^−^ ions can be adsorbed, are present on the side of the membrane facing the aqueous solution. They are schematically described by the following equations:

1 $${-}{\text{PO}}^{ (- )} + {\text{H}}^{ (+ )} \rightleftarrows {-}{\text{PO}}^{ (- )} {\text{H}}^{( + )}$$

2 $${-}{\mathop{\text{N}}\limits^{(+)}} ({\text{CH}}_{3})_{ 3} + {\text{OH}}^{(-)} \rightleftarrows {-}{\mathop{\text{N}}\limits^{(+)}} ({\text{CH}}_{3})_{3} {\text{OH}}^{(-)}$$

3 $${-}{\text{COO}}^{(-)} + {\text{H}}^{(+)} \rightleftarrows {-}{\text{COO}}^{(-)} {\text{H}}^{{({+)}}}$$

4 $${-}{\mathop {\text{N}}\limits^{(+)}} {\text{H}}_{3} + {\text{OH}}^{(-)} \rightleftarrows {-}{\mathop {\text{N}}\limits^{(+)}} {\text{H}}_{ 3} {\text{OH}}^{(-)}$$

Denoting –PO^(−)^ group as ${\text{A}}_{1}^{ - }$, –PO^(−)^H^(+)^ group as A_1_H, –N^(+)^(CH_3_)_3_ group as ${\text{B}}_{1}^{ + }$, –N^(+)^(CH_3_)_3_OH^(−)^ group as B_1_OH, –COO^(−)^ group as ${\text{A}}_{2}^{ - }$, –COO^(−)^H^(+)^ group as A_2_H, –N^(+)^H_3_ group as ${\text{B}}_{2}^{ + }$, and –N^(+)^H_3_OH^(−)^ group as B_2_OH , the above acid–base equilibria can be written in the form (Petelska and Figaszewski 2006)

5 $${\text{A}}_{1}^{ - } + {\text{H}}^{ + } \rightleftarrows {\text{A}}_{ 1} {\text{H}}$$

6 $${\text{B}}_{1}^{ + } + {\text{OH}}^{ - } \rightleftarrows {\text{B}}_{ 1} {\text{OH}}$$

7 $${\text{A}}_{2}^{ - } + {\text{H}}^{ + } \rightleftarrows {\text{A}}_{ 2} {\text{H}}$$

8 $${\text{B}}_{2}^{ + } + {\text{OH}}^{ - } \rightleftarrows {\text{B}}_{2} {\text{OH}}$$

Thus, the eight groups of active centers— ${\text{A}}_{1}^{ - }$, A_1_H, ${\text{B}}_{1}^{ + }$, B_1_OH, ${\text{A}}_{2}^{ - }$, A_2_H, ${\text{B}}_{2}^{ + }$, and B_2_OH—are present at the bilayer surface.

The surface concentration of the lipid is equal to the total number of lipids regardless of headgroup per unit area. The surface concentrations of the active center ions ($a_{{{\text{A}}_{1}^{ - } }} ,\;a_{{{\text{A}}_{1} {\text{H}}}} ,\;a_{{{\text{B}}_{1}^{ + } }} ,\;a_{{{\text{B}}_{ 1} {\text{OH}}}} ,\;a_{{{\text{A}}_{2}^{ - } }} ,\;a_{{{\text{A}}_{2} {\text{H}}}} ,\;a_{{{\text{B}}_{2}^{ + } }} ,a_{{{\text{B}}_{ 2} {\text{OH}}}}$) and the volume concentrations of the ions ($a_{{{\text{H}}^{ + } }} ,\;a_{{{\text{OH}}^{ - } }}$) determine the acid–base constants according to the relationships (Petelska and Figaszewski 2006)

9 $$K_{{{\text{A}}_{1} }} = \frac{{a_{{{\text{A}}_{ 1} {\text{H}}}} }}{{a_{{{\text{A}}_{ 1}^{ - } }} \cdot a_{{{\text{H}}^{ + } }} }}$$

10 $$K_{{{\text{B}}_{1} }} = \frac{{a_{{{\text{B}}_{ 1} {\text{OH}}}} }}{{a_{{{\text{B}}_{1}^{ + } }} \cdot a_{{{\text{OH}}^{ - } }} }}$$

11 $$K_{{{\text{A}}_{2} }} = \frac{{a_{{{\text{A}}_{ 2} {\text{H}}}} }}{{a_{{{\text{A}}_{2}^{ - } }} \cdot a_{{{\text{H}}^{ + } }} }}$$

12 $$K_{{{\text{B}}_{2} }} = \frac{{a_{{{\text{B}}_{ 2} {\text{OH}}}} }}{{a_{{{\text{B}}_{2}^{ + } }} \cdot a_{{{\text{OH}}^{ - } }} }}$$

The lipid surface concentration, denoted by *s*, can be written as (statement correct only for the 1:1 mixture)

13 $$a_{{{\text{A}}_{1}^{ - } }} + a_{{{\text{A}}_{ 1} {\text{H}}}} = \frac{s}{2}$$

14 $$a_{{{\text{B}}_{1}^{ + } }} + a_{{{\text{B}}_{1} {\text{OH}}}} = \frac{s}{2}$$

15 $$a_{{{\text{A}}_{2}^{ - } }} + a_{{{\text{A}}_{ 2} {\text{H}}}} = \frac{s}{2}$$

16 $$a_{{{\text{B}}_{2}^{ + } }} + a_{{{\text{B}}_{2} {\text{OH}}}} = \frac{s}{2}$$

Assuming additivity of the contributions of each active center to the electrical capacitance of membrane *C*_m_, the following equation can be presented:

17 $$C_{\text{m}} = C_{{{\text{A}}_{1}^{ - } }} + C_{{{\text{A}}_{ 1} {\text{H}}}} + C_{{{\text{B}}_{1}^{ + } }} + C_{{{\text{B}}_{ 1} {\text{OH}}}} + C_{{{\text{A}}_{2}^{ - } }} + C_{{{\text{A}}_{2} {\text{H}}}} + C_{{{\text{B}}_{2}^{ + } }} + C_{{{\text{B}}_{2} {\text{OH}}}}$$

The expressions describing the contributions of individual active centers to the electrical capacitance are as follows:

18 $$C_{{{\text{A}}_{1}^{ - } }} = C_{{{\text{A}}_{1}^{ - } }}^{0} \cdot \frac{{a_{{{\text{A}}_{1}^{ - } }} }}{s}$$

19 $$C_{{{\text{A}}_{1} {\text{H}}}} = C_{{{\text{A}}_{1} {\text{H}}}}^{0} \cdot \frac{{a_{{A_{1} {\text{H}}}} }}{s}$$

20 $$C_{{{\text{B}}_{1}^{ + } }} = C_{{{\text{B}}_{1}^{ + } }}^{0} \cdot \frac{{a_{{{\text{B}}_{1}^{ + } }} }}{s}$$

21 $$C_{{{\text{B}}_{1} {\text{OH}}}} = C_{{{\text{B}}_{1} {\text{OH}}}}^{0} \cdot \frac{{a_{{{\text{B}}_{1} {\text{OH}}}} }}{s}$$

22 $$C_{{{\text{A}}_{2}^{ - } }} = C_{{{\text{A}}_{2}^{ - } }}^{0} \cdot \frac{{a_{{{\text{A}}_{2}^{ - } }} }}{s}$$

23 $$C_{{{\text{A}}_{2} {\text{H}}}} = C_{{{\text{A}}_{2} {\text{H}}}}^{0} \cdot \frac{{a_{{{\text{A}}_{2} {\text{H}}}} }}{s}$$

24 $$C_{{{\text{B}}_{2}^{ + } }} = C_{{{\text{B}}_{2}^{ + } }}^{0} \cdot \frac{{a_{{{\text{B}}_{2}^{ + } }} }}{s}$$

25 $$C_{{{\text{B}}_{2} {\text{OH}}}} = C_{{{\text{B}}_{2} {\text{OH}}}}^{0} \cdot \frac{{a_{{{\text{B}}_{2} {\text{OH}}}} }}{s}$$

where $C_{{{\text{A}}_{1}^{ - } }}^{0} ,\;C_{{{\text{A}}_{1} {\text{H}}}}^{0} ,\;C_{{{\text{B}}_{1}^{ + } }}^{0} ,\;C_{{{\text{B}}_{1} {\text{OH}}}}^{0} ,\;C_{{{\text{A}}_{2}^{ - } }}^{0} ,\;C_{{{\text{A}}_{2} {\text{H}}}}^{0} ,\;C_{{{\text{B}}_{2}^{ + } }}^{0} ,\;C_{{{\text{B}}_{2} {\text{OH}}}}^{0}$ (μF cm^−2^) are the specific capacitances of the active centers.

Elimination of $a_{{{\text{A}}_{1}^{ - } }} ,\;a_{{{\text{A}}_{1} {\text{H}}}} ,\;a_{{{\text{B}}_{1}^{ + } }} ,\;a_{{{\text{B}}_{ 1} {\text{OH}}}} ,\;a_{{{\text{A}}_{2}^{ - } }} ,\;a_{{{\text{A}}_{2} {\text{H}}}} ,\;a_{{{\text{B}}_{2}^{ + } }} ,a_{{{\text{B}}_{ 2} {\text{OH}}}}$ from the equation system (9)–(25) yields

26 $$C_{\text{m}} = C_{{{\text{A}}_{1}^{ - } }}^{0} \left( {\frac{1}{{1 + K_{{{\text{A}}_{1} }} a_{{{\text{H}}^{ + } }} }}} \right) + C_{{{\text{A}}_{1} {\text{H}}}}^{0} \left( {\frac{{K_{{{\text{A}}_{1} }} a_{{{\text{H}}^{ + } }} }}{{1 + K_{{{\text{A}}_{1} }} a_{{{\text{H}}^{ + } }} }}} \right) + C_{{{\text{B}}_{1}^{ + } }}^{0} \left( {\frac{1}{{1 + K_{{{\text{B}}_{1} }} a_{{{\text{OH}}^{ - } }} }}} \right) + C_{{{\text{B}}_{1} {\text{OH}}}}^{0} \left( {\frac{{K_{{{\text{B}}_{1} }} a_{{{\text{OH}}^{ - } }} }}{{1 + K_{{{\text{B}}_{1} }} a_{{{\text{OH}}^{ - } }} }}} \right) + C_{{{\text{A}}_{2}^{ - } }}^{0} \left( {\frac{1}{{1 + K_{{{\text{A}}_{2} }} a_{{{\text{H}}^{ + } }} }}} \right) + C_{{{\text{A}}_{2} {\text{H}}}}^{0} \left( {\frac{{K_{{{\text{A}}_{2} }} a_{{{\text{H}}^{ + } }} }}{{1 + K_{{{\text{A}}_{2} }} a_{{{\text{H}}^{ + } }} }}} \right) + C_{{{\text{B}}_{2}^{ + } }}^{0} \left( {\frac{1}{{1 + K_{{{\text{B}}_{2} }} a_{{{\text{OH}}^{ - } }} }}} \right) + C_{{{\text{B}}_{2} {\text{OH}}}}^{0} \left( {\frac{{K_{{{\text{B}}_{2} }} a_{{{\text{OH}}^{ - } }} }}{{1 + K_{{{\text{B}}_{2} }} a_{{{\text{OH}}^{ - } }} }}} \right)$$

Equation (26) describes the dependence of the electrical capacitance of the 1:1 PC–PS membrane on the pH of the electrolyte solution. Theoretical values of *C*_m_ can be determined from the values of the equilibrium constants for the adsorption of H^+^ and OH^−^ on PC and PS membranes, given in (Petelska and Figaszewski 2000, 2002a); H^+^ and OH^−^ activities are calculated from the pH, and the capacitance values for individual components of the PC–PS bilayer are determined by the linear regression method. Then, Eq. (26) can be used to compare the calculated values against the experimental results. Good agreement between them will mean that the system is well described by the above equations.

## Materials and Experimental Details

### Chemicals and Preparation of the Forming Solutions

The lipid bilayer was formed from the Sigma production (St. Louis, MO, USA) of 99 % egg yolk PC and from 98 % sheep brain PS produced by Fluka (Neu-Ulm, Germany).

Both substances were dissolved in chloroform to prevent oxidation and mixed in appropriate proportions to achieve the desired molar fraction (1:1). The solvent was evaporated under a stream of argon. The dried residues were dissolved in a hexadecane–butanol mixture (10:1 by volume). The resultant solution used to form the model membrane containing 20 mg ml^−1^ of substances in solution. This solution containing the membrane components was unsaturated; therefore, it contained any proportion of the components. During membrane formation, the solvent mixture was removed, and the created membrane had the same proportion as that in the resultant solution. The samples were stored for at least 5 days at 4 °C before examination. The preparation and storage methods provided reproducible electrochemical properties when samples prepared at different times were examined using impedance method.

The solvents were of chromatographic standard purity grade; chloroform and butanol were obtained from Aldrich, and hexadecane was purchased from Fluka.

The acetate buffer was used as the electrolyte in the pH range of 2.52–7.0. It was prepared by mixing the solutions of 0.1 M acetic acid and of 0.1 M sodium acetate in appropriate proportions to achieve the required pH. The water used was triply distilled (second distillation was made with KMnO_4_ and KOH to remove organic impurities).

The experiments were performed at a temperature of about 293 ± 1 K.

### Preparation of the Bilayer Membranes

Bilayer membranes were obtained as bubbles at the Teflon cap constituting a measuring vessel component. The use of n-hexadecane as a solvent made it possible to obtain membranes with thickness and capacity values similar to values determined from studies of bilayer membranes formed from lipid monolayers (Montal and Mueller 1972). The small quantity of n-butanol had a negligible effect on the electrical parameters of the bilayers, yet it considerably accelerated membrane formation.

Thinning of the membranes was monitored using reflected light microscopy with a high-brightness yellow LED source. The microscope and the LED were mounted on supports enabling placement of the illuminator, measuring vessel, and microscope on the optical axis. The distance of the microscope from the measuring cell could also be adjusted in order to focus on the membrane located deep within the vessel.

Bilayer formation was also monitored electrically by measuring the membrane capacitance at low frequency. The capacity of the membranes increased with time after bilayer’s formation until a steady-state value was reached some 5–10 min later. The measurements were started 10–15 min after the membranes turned completely black. When the capacitance stabilized, it was assumed that diffusion of solvent out of the bilayer was complete, although some hexadecane molecules might remain “dissolved” in the membrane interior.

Membrane images were captured with a color CCD camera using the WinFast PVR program (http://winfastpvr.software.informer.com). The bilayer areas were calculated from the photographs, taking into consideration the circular nature of the surface and using the equations provided in (Bronsztejn and Siemiendiajew 1996). The area of the bilayer membranes was about 6 × 10^−2^ cm^2^.

### Electrochemical Impedance Spectroscopy and Modeling

The general architecture of the system used for electrochemical measurements was shown in (Naumowicz and Figaszewski 2011; Naumowicz et al. 2011). The setup included a personal computer, a two-phase lock-in amplifier (EG&G, Princeton Applied Research, model 5210), and a potentiostat/galvanostat (EG&G, Princeton Applied Research, model 273A), in which a four-electrode input was applied within the self-constructed electrometer.

The electrochemical cell used for impedance measurement with a bilayer lipid membrane system was described in detail (Naumowicz and Figaszewski 2014) and was placed in a Faraday cage during the measurement in order to decrease the background noise. The electrochemical cell contained two identical reversible silver–silver chloride electrodes with a salt bridge (*RE*_1_ and *RE*_2_) and two identical current platinum electrodes (*CE*_1_ and *CE*_2_). The four-electrode potentiostat assured passage of current between the two platinum electrodes in such a manner as to hold constant amplitude of voltage between the two reversible electrodes and measured intensity and phase of current in the circuit *CE*_1_–*CE*_2_. The use of the four-electrode system in the studies of electrical phenomena occurring in membranes makes it possible to considerably reduce the errors caused by electrode and electrolyte impedance (Figaszewski 1982).

Electrochemical impedance software, Power Sine 2.4, was used to carry out impedance measurements between 10 mHz and 10 kHz. The AC amplitude voltage used for the experiments was 4 mV. The impedance spectra were further analyzed by ZSimpWin 3.21 (Princeton Applied Research). The fitting process was iterative with the Chi-square (χ^2^) being used to determine the percentage error for each circuit component. The circuit elements were chosen on the basis of theories from electrochemical cell studies and using the Boukamp suggestion that each component addition should reduce the χ^2^ value by one order of magnitude. The χ^2^ value was minimized when the experimental data points correlated with the theoretical data points. This was performed by first calculating the difference between the experimental and calculated data points. The difference was squared to give larger variances a greater significance. The differences for all data points were summed and then divided by a weighing factor. According to the literature (Cui and Martin 2003), a χ^2^ value on the order of 1 × 10^−3^ or below was acceptable for a given model. The χ^2^ value was calculated by means of the procedure described in (Naumowicz et al. 2009).

## Results and Discussion

The effect of pH on the capacitance of the bilayer formed by the 1:1 PC–PS system was examined using EIS, with acetate buffer as the electrolyte. The pH of the electrolyte solution was carefully controlled during the measurements in the range from pH 2.52 to 7.0. Although the bilayer formation was only sufficiently stable for measurements within this pH range, this range was of greatest interest, given that it covers the pH of the isoelectric point of the analyzed membrane. The sensitivity of the thermotropic phase of PS model membranes to variations in pH was characterized (Cevc and Marsh 1987; Cevc et al. 1981; MacDonald et al. 1976). The gel/liquid crystalline phase-transition temperature of the PS bilayer was very sensitive to pH, over pH ranges corresponding to the ionization of the phosphate (pH ~1–2), carboxylate (pH ~3.5–4.5), and amino (pH ~9.5–10.5) moieties of the polar headgroup. The highest phase transition was found for the zwitterionic form of PS below pH ~2, where electrostatic PS repulsion and hydration of the polar headgroup were minimal.

The impedance technique was used to characterize the membrane features, since this method has shown to be able to measure the membrane capacitance of bilayer lipid membranes accurately (Coster 2003). Each value of the measured parameters was calculated as an arithmetic mean and standard deviation for at least six membranes. The experimental impedance values are related to the bilayer surface area unit. Based on our experimental results and numerous literature data (Coster 2003; Karolins et al. 1998), we assume that our membranes do not retain solvent. However, if a small fraction is retained in the membrane then solvent molecules can be treated as impurities. Since it is impossible to determine the quantity of these impurities, it is impossible to make a thorough qualitative determination of their nature and so one cannot take them into account in quantitative considerations (except for a possible qualitative indication). If quantitative analysis was possible, then we would take into account the possible presence of any solvent in the derived equations.

**Fig. 1:**
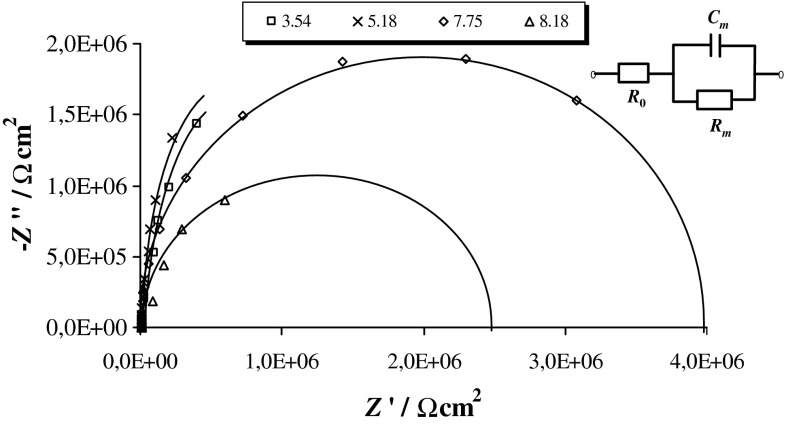
Complex plane impedance diagrams of phosphatidylcholine–phosphatidylserine bilayers registered at different pH values. The solid lines represent the results of the fitting procedure. The equivalent circuit used for impedance data analysis is shown in the inset: *R*_0_ represents the resistance of the electrolyte, *R*_m_—the resistance of the membrane, and *C*_m_—the capacitance of the membrane

Figure 1 shows typical impedance spectra of the PC–PS membranes formed at different pH values. The spectra for some pH values have been omitted for clarity. Very simple diagrams were obtained for all of the examined membranes. These diagrams have the form of semicircles or arcs in the entire range of analyzed frequencies. The centers of the semicircles lie on the real axis, provided that the lipid bilayers are considered as dielectric layers with leakage.

The equivalent circuit used for data analysis (inset in Fig. 1) consists of a parallel arrangement of the capacitor *C*_m_ and resistor *R*_m_, attributed to the electrical properties of the bilayer, completed by a serial resistor *R*_0_ for the bulk conductivity. Values of *C*_m_ and *R*_m_ were calculated from the experimental complex impedance after subtraction of *R*_0_. Then, they were recalculated as a set of parallel components and normalized for membrane area. The electrochemical parameters of the circuit were evaluated by employing the ZsimpWin software. A very high correlation was observed between the experimental results and the results calculated with the best-fitting electrical equivalent circuit model, where χ^2^ was minimized to ≤10^−3^. An examination of the data obtained for the analyzed systems indicated that the proposed equivalent circuit can be used to describe the experimental results.

**Fig. 2:**
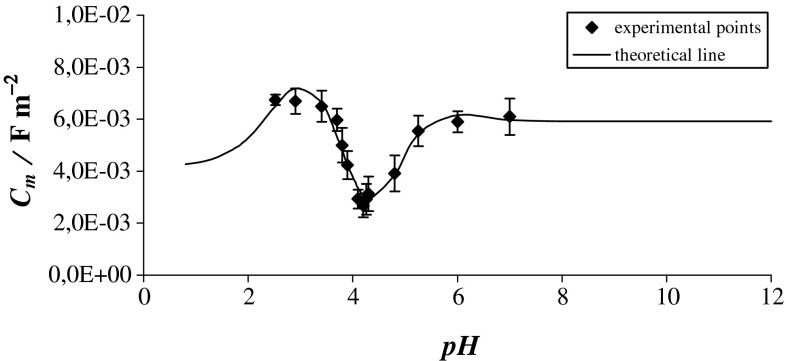
The dependence of the electrical capacitance *C*_m_ of a bilayer membrane formed from PC–PS system on the pH of the electrolyte solution

Figure 2 shows the plot of the electrical capacitance of the bilayer formed by the PC–PS system against the pH of the electrolyte solution. Experimental values are presented by points, and the theoretical values calculated from Eq. (26) are marked with a continuous line. This figure refers to the above-described structural model of the PC–PS membrane surface, in which the functional groups were assumed to be uniformly distributed on the surface from the aqueous solution side.

The minimum capacitance, equal to 0.268 ± 0.036 μF cm^−2^ at pH 4.20, was obtained when the pH approached the isoelectric point. This point corresponds to the pH at which the surface formed by the PC–PS system has no net electrical charge, or where the negative and positive charges are equal. The capacitance of the PC–PS bilayer increases asymmetrically, whereas the capacitance for a pure PC bilayer was shown to increase symmetrically toward low or high pH (Naumowicz et al. 2013). This may be due to the asymmetrical dissociable groups of PS (one positive and two negative ionizable groups), whereas PC has a zwitterionic group (positive and negative ionized groups). Alternatively, the change in capacitance with the change in pH may be caused by the interaction of the PC–PS bilayer with solution components other than H^+^ and OH^−^ (i.e., acetic acid and sodium acetate in the electrolyte), which were not accounted for in Eq. (26). It may be that ions other than H^+^ and OH^−^, such as acetate, are adsorbed at the PC–PS membrane.

Based on Eq. (26), the total capacitance value of the PC–PS membrane is the sum of the capacitances of its components, i.e., ${\text{A}}_{1}^{ - }$, A_1_H, ${\text{B}}_{1}^{ + }$, B_1_OH, ${\text{A}}_{2}^{ - }$, A_2_H, ${\text{B}}_{2}^{ + }$ , and B_2_OH. To calculate the values of the specific capacitance of these components, the equilibrium constants of adsorption processes of H^+^ and OH^−^ on PC and PS must be known. Acid–base equilibrium constants were determined by titration of PC and PS liposomes with HCl and NaOH (Petelska and Figaszewski 2000, 2002a). The use of liposomes ensures a uniform distribution of acidic and basic groups, in spite of the water insolubility of phospholipids. For PC, *K*_A1_ = 10^2.581^ and *K*_B1_ = 10^5.687^, where *K*_A1_ is assigned to the –PO^(−)^ group and *K*_B1_ to the –N^(+)^(CH_3_)_3_ group (Petelska and Figaszewski 2000). Acid–base equilibrium constants for the PS membrane are *K*_A1_ = 10^2.581^, *K*_A2_ = 10^4.139^, and *K*_B2_ = 10^9.55^ for the –PO^(−)^, –COO^(−)^, and –N^(+)^H_3_ groups, respectively (Petelska and Figaszewski 2002a). The *K*_A1_, *K*_A2_, *K*_B1_, and *K*_B2_ values were substituted into Eq. (26) to calculate the electrical capacitance values at different pH values.

The capacitance values for individual components of the PC–PS bilayer were determined by linear regression method. $C_{{{\text{A}}_{1}^{ - } }}^{0} ,\;C_{{{\text{A}}_{1} {\text{H}}}}^{0} ,\;C_{{{\text{B}}_{1}^{ + } }}^{0} ,\;C_{{{\text{B}}_{1} {\text{OH}}}}^{0} ,\;C_{{{\text{A}}_{2}^{ - } }}^{0} ,\;C_{{{\text{A}}_{2} {\text{H}}}}^{0} ,\;C_{{{\text{B}}_{2}^{ + } }}^{0} ,$ and $C_{{{\text{B}}_{2} {\text{OH}}}}^{0}$ were 0.0059 ± 0.0014, 0.0000 ± 0.0001, -0.0347 ± 0.0012, 0.0000 ± 0.0001, 0.0000 ± 0.0001, -0.0020 ± 0.0002, 0,0042 ± 0.0006, and 0.0367 ± 0.0007 μF cm^−2^, respectively. Thus, one can conclude that the specific capacitances of the membrane components have positive values for –PO, –NH_3_, and –NH_3_OH groups. All other ionizable groups are obviously still in the membrane but the results are possibly telling us that ionization is prevented somehow through head–head contacts. Moreover, there is a possibility of strong electrostatic attraction between hydrophobic parts and appropriate charged moieties of the polar headgroups. The electrophoretic mobility of zwitterionic lipid vesicles dispersed in salt solutions (Tatulian 1993) and nuclear magnetic resonance (NMR) measurements (Rydall and Macdonald 1992) indicated that in addition to charged lipids, ions may also bind to zwitterionic lipids. Adsorption of ions on lipid bilayers appears to be highly ion specific, because different ions with identical charges can have different binding constants and exhibit very different effects on membrane properties (Petrache et al. 2006). Anions and cations other than hydroxide and hydrogen ions were proven to have the potency of lowering the membrane dipole potential. Specifically, anions with the lowest free energy of hydration (i.e., the least hydrophilic ones) induce the greatest decrease in the dipole potential, whereas the most hydrophilic cations cause the greatest reduction in the membrane dipole potential (Clarke and Lupfert 1999). As a possible model for the opposite behavior of cations in this respect, it was proposed that they either interact with specific polar sites found on the membrane surface, they may contribute to a partial dehydration of the membrane head group region, or both (Chiriac and Luchian 2007). It should be kept in mind the possibility of the interaction of the PC–PS membrane with the solution components other than H^+^ and OH^−^. The electrolyte contained acetic acid and sodium acetate. The interaction with those electrolyte solution components was not taken into account during theoretical considerations; perhaps other ions like acetate or sodium are adsorbed at the analyzed membrane in addition to the H^+^ and OH^−^ ions.

Besides, three centers in PS headgroups at neutral pH can form hydrogen bonds to adjacent lipids. The hydrogen-bonding propensity of PS lipids has been measured using FTIR (Lewis and McElhaney 2000). Molecular dynamics simulations have emphasized hydrogen bond formation and lateral compression from DPPC to DPPS (Pandit and Berkowitz 2002). Lateral condensation has been shown to involve hydrogen bond formation between the ${\text{NH}}_{3}^{ + }$ and ${\text{PO}}_{4}^{ - }$ groups, as well as binding of Na^+^ to the COO^−^ group. Moreover, the hydrogen bonds reduce the distance at which steric interactions become important when compared to the sum of van der Waals radii (Petrache et al. 2004).

The acid–base equilibrium in the PC–PS bilayer was not quantified by the values of resistance, because resistance often shows more significant scattering than capacity. Resistance is burdened with random errors caused by the presence of the solvent and ions from the electrolyte solution in the bilayer. The presence of these ions and the solvent always leads to a meaningful, and easily noticeable error and, therefore, scattering in the results. This effect does not occur in such a visually perceptible manner in the capacity. For these reasons, results based on resistance measurements are often treated as supplementary data.

**Fig. 3:**
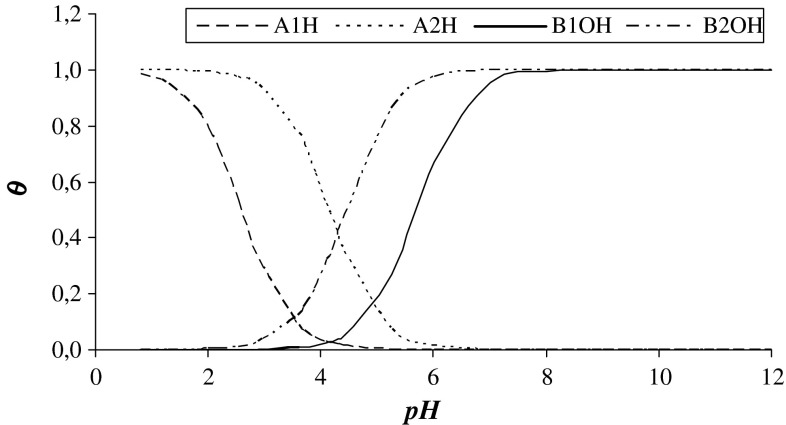
The degree of coverage of the phosphatidylcholine–phosphatidylserine bilayer surface with associated forms A_1_H, B_1_OH, A_2_H, and B_2_OH groups as a function of pH of the electrolyte solution

Figure 3 demonstrates the degree of coverage of the PC–PS membrane surface by associated and dissociated forms of the groups present at the membrane surface as a function of the pH of the electrolyte solution. The membrane surface was almost not covered by H^+^ and OH^−^ when the pH was near the isoelectric point (i.e., pH 4.2).

The effect of pH on the behavior of amphiphilic substances at the air–water interface was first examined at the beginning of the last century (Schulman and Hughes 1932), in the form of surface potential investigations at fixed values of area/molecule. The pH and ionic composition of the aqueous medium and the membrane surface charge are the important parameters influencing membrane organization. For example, the structure of aqueous dispersions of negatively charged PS depends markedly on the pH and salt concentration (particularly, the concentration of divalent cations), which determine whether the molecules will arrange in lamellar or nonlamellar phases (Tessier et al. 2004).

Various techniques, such as Langmuir film balance (Petriat and Giasson 2005) and NMR (Sulkowski et al. 2005), electron paramagnetic resonance (Sulkowski et al. 2005) and fluorescence spectroscopy (Furuike et al. 1999), have documented changes in many membrane properties, including liposome stability, lateral phase separation, and the interdigitated gel-to-bilayer gel phase transition, in response to changes in pH (Furuike et al. 1999; Petriat and Giasson 2005; Sulkowski et al. 2005). In particular, calorimetric studies have established that low pH environments (pH ≤2) can increase PC membrane phase-transition temperatures (Stumpel et al. 1980). The effect of pH on membrane interfacial tension has been studied by measuring the curvature change of a phospholipid drop exposed to different pH environments (Petelska and Figaszewski 2000, 2002a, b, 2006). The interfacial tension of PC (Petelska and Figaszewski 2000, 2002a), PS (Petelska and Figaszewski 2002a), PC–PS (Petelska and Figaszewski 2006), and PE (Petelska and Figaszewski 2002b) exhibited maximal values at the corresponding isoelectric points—pH 4.12 for PC, pH 3.80 for PS, pH 4 for PC–PS, and pH 4.18 for PE—similar to that observed for biological membranes (Wojtczak and Nałęcz 1979).

The results presented in this paper are compatible to data reported by Petelska and Figaszewski. Zhou and Raphael (2007) demonstrated that the solution pH affects both the membrane mechanical and interfacial electrical properties, and that alterations in membrane surface charge density, and the Debye length can account for the experimentally measured changes in the membrane bending stiffness.

Another paper (Ohki 1969) observed the effect of variations in pH on the capacitance of pure PC and pure PS bilayers. The capacitance of the bilayers was larger in a solution of lower or higher pH than in a solution of medium pH. Since PC has a zwitterionic group and PS has three dissociable polar groups, the polar groups would be positively or negatively charged at lower or higher pH values. Dissociation of the phosphate groups occurs in these pH ranges in the bilayer state, which corresponds to the minimum value of the capacitance. In the present paper, a minimum corresponding to the isoelectric point appeared in both the theoretical equation and in the experimental data.

## Conclusions

In conclusion, noninvasive EIS method was used to characterize the capacitive properties of the PC–PS bilayer and to provide a quantitative description of the acid–base equilibria at the interface separating the electrolyte solution and bilayer. The electrical capacitance of the analyzed bilayer had a minimum value around pH 4.2. The value of the capacitance increased as the pH of the solution decreased or increased.

Therefore, pH appears to play an important role in setting the electrical properties of bilayer lipid membranes. This information may be useful for investigating the functional properties of lipid membranes and membrane proteins under aqueous pH stress.
